# Vagus Nerve as Modulator of the Brain–Gut Axis in Psychiatric and Inflammatory Disorders

**DOI:** 10.3389/fpsyt.2018.00044

**Published:** 2018-03-13

**Authors:** Sigrid Breit, Aleksandra Kupferberg, Gerhard Rogler, Gregor Hasler

**Affiliations:** ^1^Division of Molecular Psychiatry, Translational Research Center, University Hospital of Psychiatry, University of Bern, Bern, Switzerland; ^2^Department of Gastroenterology and Hepatology, University Hospital Zurich, Zurich, Switzerland

**Keywords:** depression, PTSD, vagus nerve stimulation, nutrition, probiotics, yoga, meditation, inflammatory bowel disease

## Abstract

The vagus nerve represents the main component of the parasympathetic nervous system, which oversees a vast array of crucial bodily functions, including control of mood, immune response, digestion, and heart rate. It establishes one of the connections between the brain and the gastrointestinal tract and sends information about the state of the inner organs to the brain *via* afferent fibers. In this review article, we discuss various functions of the vagus nerve which make it an attractive target in treating psychiatric and gastrointestinal disorders. There is preliminary evidence that vagus nerve stimulation is a promising add-on treatment for treatment-refractory depression, posttraumatic stress disorder, and inflammatory bowel disease. Treatments that target the vagus nerve increase the vagal tone and inhibit cytokine production. Both are important mechanism of resiliency. The stimulation of vagal afferent fibers in the gut influences monoaminergic brain systems in the brain stem that play crucial roles in major psychiatric conditions, such as mood and anxiety disorders. In line, there is preliminary evidence for gut bacteria to have beneficial effect on mood and anxiety, partly by affecting the activity of the vagus nerve. Since, the vagal tone is correlated with capacity to regulate stress responses and can be influenced by breathing, its increase through meditation and yoga likely contribute to resilience and the mitigation of mood and anxiety symptoms.

## Introduction

The bidirectional communication between the brain and the gastrointestinal tract, the so-called “brain–gut axis,” is based on a complex system, including the vagus nerve, but also sympathetic (e.g., *via* the prevertebral ganglia), endocrine, immune, and humoral links as well as the influence of gut microbiota in order to regulate gastrointestinal homeostasis and to connect emotional and cognitive areas of the brain with gut functions (1). The ENS produces more than 30 neurotransmitters and has more neurons than the spine. Hormones and peptides that the ENS releases into the blood circulation cross the blood–brain barrier (e.g., ghrelin) and can act synergistically with the vagus nerve, for example to regulate food intake and appetite (2). The brain–gut axis is becoming increasingly important as a therapeutic target for gastrointestinal and psychiatric disorders, such as inflammatory bowel disease (IBD) (3), depression (4), and posttraumatic stress disorder (PTSD) (5). The gut is an important control center of the immune system and the vagus nerve has immunomodulatory properties (6). As a result, this nerve plays important roles in the relationship between the gut, the brain, and inflammation. There are new treatment options for modulating the brain–gut axis, for example, vagus nerve stimulation (VNS) and meditation techniques. These treatments have been shown to be beneficial in mood and anxiety disorders (7–9), but also in other conditions associated with increased inflammation (10). In particular, gut-directed hypnotherapy was shown to be effective in both, irritable bowel syndrome and IBD (11, 12). Finally, the vagus nerve also represents an important link between nutrition and psychiatric, neurological and inflammatory diseases.

## Basic Anatomy of the Vagus Nerve

The vagus nerve carries an extensive range of signals from digestive system and organs to the brain and *vice versa*. It is the tenth cranial nerve, extending from its origin in the brainstem through the neck and the thorax down to the abdomen. Because of its long path through the human body, it has also been described as the “wanderer nerve” (13).

**Figure 1:**
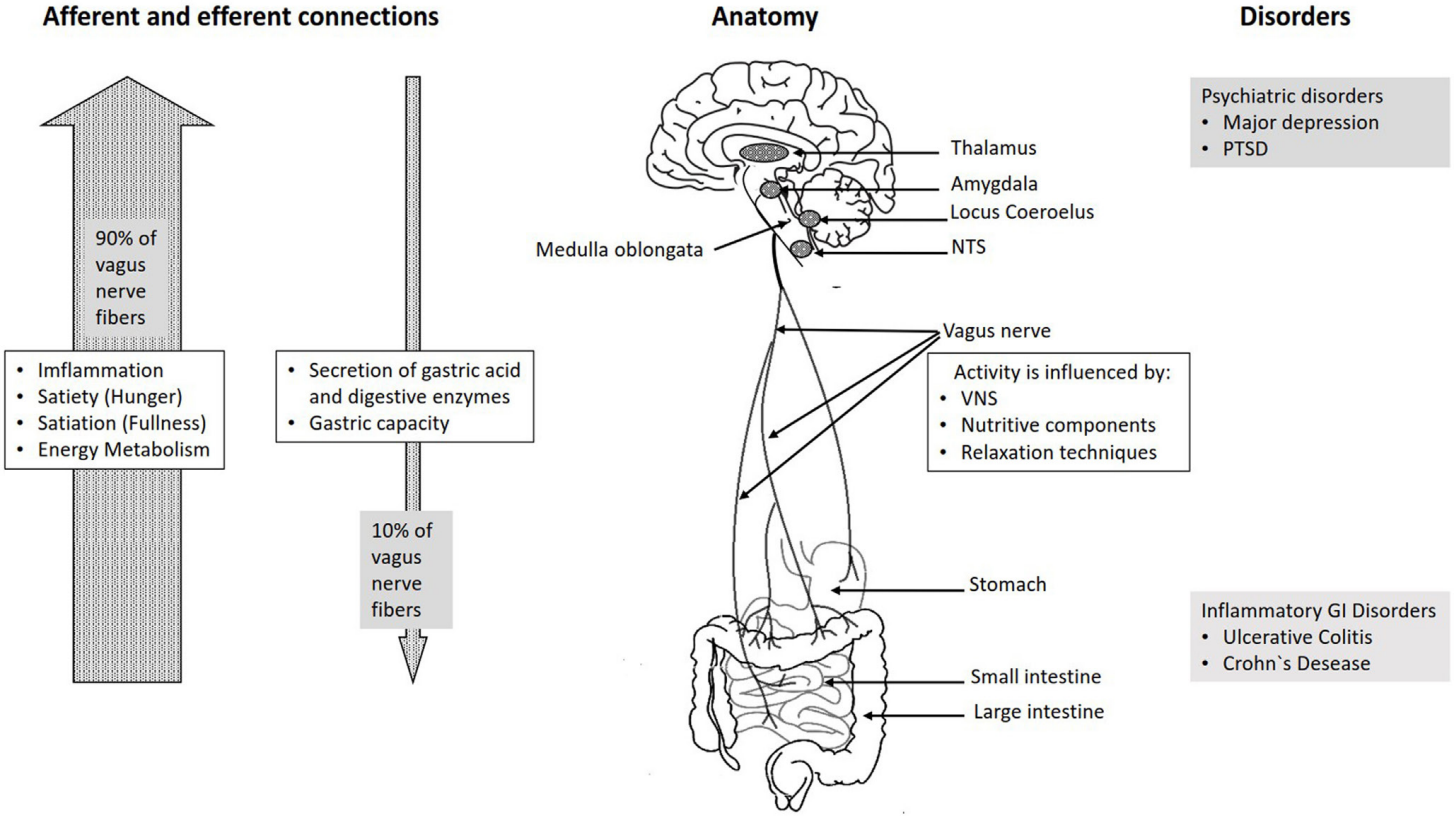
Overview over the basic anatomy and functions of the vagus nerve.

The vagus nerve exits from the medulla oblongata in the groove between the olive and the inferior cerebellar peduncle, leaving the skull through the middle compartment of the jugular foramen. In the neck, the vagus nerve provides required innervation to most of the muscles of the pharynx and larynx, which are responsible for swallowing and vocalization. In the thorax, it provides the main parasympathetic supply to the heart and stimulates a reduction in the heart rate. In the intestines, the vagus nerve regulates the contraction of smooth muscles and glandular secretion. Preganglionic neurons of vagal efferent fibers emerge from the dorsal motor nucleus of the vagus nerve located in the medulla, and innervate the muscular and mucosal layers of the gut both in the lamina propria and in the muscularis externa (14). The celiac branch supplies the intestine from proximal duodenum to the distal part of the descending colon (15, 16). The abdominal vagal afferents, include mucosal mechanoreceptors, chemoreceptors, and tension receptors in the esophagus, stomach, and proximal small intestine, and sensory endings in the liver and pancreas. The sensory afferent cell bodies are located in nodose ganglia and send information to the nucleus tractus solitarii (NTS) (see Figure 1). The NTS projects, the vagal sensory information to several regions of the CNS, such as the locus coeruleus (LC), the rostral ventrolateral medulla, the amygdala, and the thalamus (14).

The vagus nerve is responsible for the regulation of internal organ functions, such as digestion, heart rate, and respiratory rate, as well as vasomotor activity, and certain reflex actions, such as coughing, sneezing, swallowing, and vomiting (17). Its activation leads to the release of acetylcholine (ACh) at the synaptic junction with secreting cells, intrinsic nervous fibers, and smooth muscles (18). ACh binds to nicotinic and muscarinic receptors and stimulates muscle contractions in the parasympathetic nervous system.

Animal studies have demonstrated a remarkable regeneration capacity of the vagus nerve. For example, subdiaphragmatic vagotomy induced transient withdrawal and restoration of central vagal afferents as well as synaptic plasticity in the NTS (19). Further, the regeneration of vagal afferents in rats can be reached 18 weeks after subdiaphragmatic vagotomy (20), even though the efferent reinnervation of the gastrointestinal tract is not restored even after 45 weeks (21).

## Functions of the Vagus Nerve

### The Role of Vagus in the Functions of the Autonomic Nervous System

Alongside the sympathetic nervous system and the enteric nervous system (ENS), the parasympathetic nervous system represents one of the three branches of the autonomic nervous system.

The definition of the sympathetic and parasympathetic nervous systems is primarily anatomical. The vagus nerve is the main contributor of the parasympathetic nervous system. Other three parasympathetic cranial nerves are the nervus oculomotorius, the nervus facialis, and the nervus glossopharyngeus.

The most important function of the vagus nerve is afferent, bringing information of the inner organs, such as gut, liver, heart, and lungs to the brain. This suggests that the inner organs are major sources of sensory information to the brain. The gut as the largest surface toward the outer world and might, therefore, be a particularly important sensory organ.

Historically, the vagus has been studied as an efferent nerve and as an antagonist of the sympathetic nervous system. Most organs receive parasympathetic efferents through the vagus nerve and sympathetic efferents through the splanchnic nerves. Together with the sympathetic nervous systems, the parasympathetic nervous system is responsible for the regulation of vegetative functions by acting in opposition to each other (22). The parasympathetic innervation causes a dilatation of blood vessels and bronchioles and a stimulation of salivary glands. On the contrary, the sympathetic innervation leads to a constriction of blood vessels, a dilatation of bronchioles, an increase in heart rate, and a constriction of intestinal and urinary sphincters. In the gastrointestinal tract, the activation of the parasympathetic nervous system increases bowel motility and glandular secretion. In contrast to it, the sympathetic activity leads to a reduction of intestinal activity and a reduction of blood flow to the gut, allowing a higher blood flow to the heart and the muscles, when the individual faces existential stress.

The ENS arises from neural crest cells of the primarily vagal origin and consists of a nerve plexus embedded in the intestinal wall, extending across the whole gastrointestinal tract from the esophagus to the anus. It is estimated that the human ENS contains about 100–500 million neurons. This is the largest accumulation of nerve cells in the human body (23–25). Since the ENS is similar to the brain regarding structure, function, and chemical coding, it has been described as “the second brain” or “the brain within the gut” (26). It consists of two ganglionated plexuses—the submucosal plexus, which regulates gastrointestinal blood flow and controls the epithelial cell functions and secretion and the myenteric plexus, which mainly regulates the relaxation and contraction of the intestinal wall (23). The ENS serves as intestinal barrier and regulates the major enteric processes, such as immune response, detecting nutrients, motility, microvascular circulation, and epithelial secretion of fluids, ions, and bioactive peptides (27). There clearly is “communication” between the vagal nerve and the ENS, and the main transmitter is cholinergic activation through nicotinic receptors (24). Interaction of ENS and the vagal nerve as a part of the CNS leads to a bidirectional flow of information. On the other hand, the ENS in the small and large bowel also is able to function quite independent of vagal control as it contains full reflex circuits, including sensory neurons and motor neurons. They regulate muscle activity and motility, fluid fluxes, mucosal blood flow, and also mucosal barrier function. ENS neurons are also in close contact to cells of the adaptive and innate immune system and regulate their functions and activities. Aging and cell loss in the ENS are associated with complaints, such as constipation, incontinence, and evacuation disorders. The loss of the ENS in the small and large intestine may be life threatening (Hirschsprung’s disease; intestinal pseudo-obstruction), whereas as loss of the vagal nerve in these areas is not.

### Vagus Nerve as a Link between the Central and ENS

The connection between the CNS and the ENS, also referred to as the brain–gut axis enables the bidirectional connection between the brain and the gastrointestinal tract. It is responsible for monitoring the physiological homeostasis and connecting the emotional and cognitive areas of the brain with peripheral intestinal functions, such as immune activation, intestinal permeability, enteric reflex, and enteroendocrine signaling (1). This brain–gut axis, includes the brain, the spinal cord, the autonomic nervous system (sympathetic, parasympathetic, and ENS), and the hypothalamic–pituitary–adrenal (HPA) axis (1). The vagal efferents send the signals “down” from brain to gut through efferent fibers, which account for 10–20% of all fibers and the vagal afferents “up” from the intestinal wall to the brain accounting for 80–90% of all fibers (28) (see Figure 1). The vagal afferent pathways are involved in the activation/regulation of the HPA axis (29), which coordinates the adaptive responses of the organism to stressors of any kind (30). Environmental stress, as well as elevated systemic proinflammatory cytokines, activates the HPA axis through secretion of the corticotropin-releasing factor (CRF) from the hypothalamus (31). The CRF release stimulates adrenocorticotropic hormone (ACTH) secretion from pituitary gland. This stimulation, in turn, leads to cortisol release from the adrenal glands. Cortisol is a major stress hormone that affects many human organs, including the brain, bones, muscles, and body fat.

Both neural (vagus) and hormonal (HPA axis) lines of communication combine to allow brain to influence the activities of intestinal functional effector cells, such as immune cells, epithelial cells, enteric neurons, smooth muscle cells, interstitial cells of Cajal, and enterochromaffin cells (32). These cells, on the other hand, are under the influence of the gut microbiota. The gut microbiota has an important impact on the brain–gut axis interacting not only locally with intestinal cells and ENS, but also by directly influencing neuroendocrine and metabolic systems (33). Emerging data support the role of microbiota in influencing anxiety and depressive-like behaviors (34). Studies conducted on germ-free animals demonstrated that microbiota influence stress reactivity and anxiety-like behavior and regulate the set point for HPA activity. Thus, these animals generally show a decreased anxiety (35) and an increased stress response with augmented levels of ACTH and cortisol (36).

In case of food intake, vagal afferents innervating the gastrointestinal tract provide a rapid and discrete account of digestible food as well as circulating and stored fuels, while vagal efferents together with the hormonal mechanisms codetermine the rate of nutrient absorption, storage, and mobilization (37). Histological and electrophysiological evidence indicates that visceral afferent endings of the vagus nerve in the intestine express a diverse array of chemical and mechanosensitive receptors. These receptors are targets of gut hormones and regulatory peptides that are released from enteroendocrine cells of the gastrointestinal system in response to nutrients, by distension of the stomach and by neuronal signals (38). They influence the control of food intake and regulation of satiety, gastric emptying and energy balance (39) by transmitting signals arising from the upper gut to the nucleus of the solitary tract in the brain (40). Most of these hormones, such as peptide cholecystokinin (CCK), ghrelin, and leptin are sensitive to the nutrient content in the gut and are involved in regulating short-term feelings of hunger and satiety (41).

Cholecystokinin regulates gastrointestinal functions, including inhibition of gastric emptying and food intake through activation of CCK-1 receptors on vagal afferent fibers innervating the gut (42). In addition, CCK is important for secretion of pancreatic fluid and producing gastric acid, contracting the gallbladder, decreasing gastric emptying, and facilitating digestion (43). Saturated fat, long-chain fatty acids, amino acids, and small peptides that result from protein digestion stimulate the release of CCK from the small intestine (44). There are various biologically active forms of CCK, classified according to the number of amino acids they contain, i.e., CCK-5, CCK-8, CCK-22, and CCK-33 (45). In neurons, CCK-8 is always the predominating form, whereas the endocrine gut cells contain a mixture of small and larger CCK peptides of which CCK-33 or CCK-22 often predominate (42). In rats, both long- and short-chain fatty acids from food activate jejunal vagal afferent nerve fibers, but do so by distinct mechanisms (46). Short-chain fatty acids, such as butyric acid have a direct effect on vagal afferent terminals while the long-chain fatty acids activate vagal afferents *via* a CCK-dependent mechanism. Exogenous administration of CCK appears to inhibit endogenous CCK secretion (47). CCK is also present in enteric vagal afferent neurons, in cerebral cortex, in the thalamus, hypothalamus, basal ganglia, and dorsal hindbrain, and functions as a neurotransmitter (45). It directly activates vagal afferent terminals in the NTS by increasing calcium release (48). Further, there is evidence that CCK can activate neurons in the hindbrain and intestinal myenteric plexus (a plexus which provides motor innervation to both layers of the muscular layer of the gut), in rats and that vagotomy or capsaicin treatment results in an attenuation of CCK-induced Fos expression (a type of a proto-oncogene) in the brain (43). There is also substantial evidence that elevated levels of CCK induce feelings of anxiety (49). Therefore, CCK is used as a challenge agent to model anxiety disorders in humans and animals (50).

Ghrelin is another hormone released into circulation from the stomach and plays a key role in stimulating food intake by inhibiting vagal afferent firing (51). Circulating ghrelin levels are increased by fasting and fall after a meal (52). Central or peripheral administration of acylated ghrelin to rats acutely stimulates food intake and growth hormone release, and chronic administration causes weight gain (53). The action of ghrelin’s on feeding is abolished or attenuated in rats that have undergone vagotomy or treatment with capsaicin, a specific afferent neurotoxin (54, 55). In humans, intravenous infusion or subcutaneous injection increases both feelings of hunger and food intake, since ghrelin suppresses insulin release (56). Therefore, it is not surprising that secretion is disturbed in obesity and insulin resistance (57).

Leptin receptors have also been identified in the vagus nerve. Studies in rodents clearly indicate that leptin and CCK interact synergistically to induce short-term inhibition of food intake and long-term reduction of body weight (40). The epithelial cells that respond to both ghrelin and leptin are located near the vagal mucosal endings and modulate the activity of vagal afferents, acting in concert to regulate food intake (58, 59). After fasting and diet-induced obesity in mice, leptin loses its potentiating effect on vagal mucosal afferents (59).

The gastrointestinal tract is the key interface between food and the human body and can sense basic tastes in much the same way as the tongue, through the use of similar G-protein-coupled taste receptors (60). Different taste qualities induce the release of different gastric peptides. Bitter taste receptors can be considered as potential targets to reduce hunger by stimulating the release of CCK (61). Further, activation of bitter taste receptors stimulates ghrelin secretion (62) and, therefore, affects the vagus nerve.

### Vagus Nerve as Modulator of Intestinal Immune Homeostasis

The gastrointestinal tract is constantly confronted with food antigens, possible pathogens, and symbiotic intestinal microbiota that present a risk factor for intestinal inflammation (63). It is highly innervated by vagal fibers that connect the CNS with the intestinal immune system, making vagus a major component, the neuroendocrine-immune axis. This axis is involved in coordinated neural, behavioral, and endocrine responses, important for the first-line defense against inflammation (64). For example, in response to pathogens and other injurious stimuli, tumor-necrosis factor-alpha (TNF-α), a cytokine, is produced by activated macrophages, dendritic cells, and other cells in the mucosa (3, 65). Together with prostaglandins and interferons, TNF-α is an important mediator of local and systemic inflammation and increases cause the cardinal clinical signs of inflammation, including heat, swelling, pain, and redness (66, 67). Counter-regulatory mechanisms, such as immunologically competent cells and anti-inflammatory cytokines normally limit the acute inflammatory response and prevent the spread of inflammatory mediators into the bloodstream. Further, there is a “hard-wired” connection between the nervous and immune system functions as an anti-inflammatory mechanism. The dorsal vagal complex, comprising the sensory nuclei of the solitary tract, the area postrema, and the dorsal motor nucleus of the vagus, responds to increased circulating amounts of TNF-α by altering motor activity in the vagus nerve (68).

The anti-inflammatory capacities of the vagus nerve are mediated through three different pathways (18). The first pathway is the HPA axis, which has been described above. The second pathway is the splenic sympathetic anti-inflammatory pathway, where the vagus nerve stimulates the splenic sympathetic nerve. Norepinephrine (NE) (noradrenaline) released at the distal end of the splenic nerve links to the β2 adrenergic receptor of splenic lymphocytes that release ACh. Finally, ACh inhibits the release of TNF-α by spleen macrophages through α-7-nicotinic ACh receptors. The last pathway, called the cholinergic anti-inflammatory pathway (CAIP), is mediated through vagal efferent fibers that synapse onto enteric neurons, which in turn release ACh at the synaptic junction with macrophages (18). ACh binds to α-7-nicotinic ACh receptors of those macrophages to inhibit the TNF-α (69). Compared to the HPA axis, the CAIP has some unique properties, such as a high speed of neural conductance, which enables an immediate modulatory input to the affected region of inflammation (70). Therefore, the CAIP plays a crucial role in the intestinal immune response and homeostasis, and presents a highly interesting target for the development of novel treatments for inflammatory diseases related to the gut immune system (6, 18).

The inflammation-sensing and inflammation-suppressing functions outlined above provide the principal components of the inflammatory reflex (71). The appearance of pathogenic organisms activates innate immune cells that release cytokines. These in turn activate sensory fibers that ascend in the vagus nerve to synapse in the nucleus tractus solitarius. Increased efferent signals in the vagus nerve suppress peripheral cytokine release through macrophage nicotinic receptors and the CAIP. Thus, experimental activation of the CAIP by direct electrical stimulation of the efferent vagus nerve inhibits the synthesis of TNF-α in liver, spleen, and heart, and attenuates serum concentrations of TNF-α (72, 73).

## Vagus Nerve Stimulation

Vagus nerve stimulation is a medical treatment that is routinely used in the treatment of epilepsy and other neurological conditions. VNS studies are not just clinically, but also scientifically informative regarding the role of the vagus nerve in health and disease.

### Device and Method

Vagus nerve stimulation works by applying electrical impulses to the vagus nerve. The stimulation of the vagus nerve can be performed in two different ways: a direct invasive stimulation, which is currently the most frequent application and an indirect transcutaneous non-invasive stimulation. Invasive VNS (iVNS) requires the surgical implantation of a small pulse generator subcutaneously in the left thoracic region. Electrodes are attached to the left cervical vagus nerve and are connected to the pulse generator by a lead, which is tunneled under the skin. The generator delivers intermittent electrical impulses through the vagus nerve to the brain (74). It is postulated that these electrical impulses exert antiepileptic (75), antidepressive (76), and anti-inflammatory effects by altering the excitability of nerve cells. In contrast to iVNS, transcutaneous VNS (tVNS) allows for a non-invasive stimulation of the vagus nerve without any surgical procedure. Here, the stimulator is usually attached to the auricular concha *via* ear clips and delivers electrical impulses at the subcutaneous course of the afferent auricular branch of the vagus nerve (77). A pilot study that examined the application of VNS in 60 patients with treatment-resistant depressive disorder showed a significant clinical improvement in 30–37% of patients and a high tolerability (78). Five years later, the stimulation of the vagus nerve for the treatment of refractory depression was approved by the U.S. Food and Drug Administration (FDA) (79). Since then, the safety and efficacy of VNS in depression has been demonstrated in numerous observational studies as can be seen below. In contrast, there is no randomized, placebo-control clinical trial that reliably demonstrates antidepressant effects of VNS.

### The Neural Mechanism of VNS

The mechanism by which VNS may benefit patients nonresponsive to conventional antidepressants is unclear, with further research needed to clarify this (80). Functional neuroimaging studies have confirmed that VNS alters the activity of many cortical and subcortical regions (81). Through direct or indirect anatomic connections *via* the NTS, the vagus nerve has structural connections with several mood regulating limbic and cortical brain areas (82). Thus, in chronic VNS for depression, PET scans showed a decline in resting brain activity in the ventromedial prefrontal cortex (vmPFC), which projects to the amygdala and other brain regions modulating emotion (83). VNS results in chemical changes in monoamine metabolism in these regions possibly resulting in antidepressant action (84, 85). The relationship between monoamine and antidepressant action has been shown by various types of evidence. All drugs that increase monoamines—serotonin (5-HT), NE, or dopamine (DA)—in the synaptic cleft have antidepressant properties (86). Accordingly, depletion of monoamines induces depressive symptoms in individuals who have an increased risk of depression (87).

Chronic VNS influences the concentration of 5-HT, NE, and DA in the brain and in the cerebrospinal fluid (88). In rats, it has been shown that VNS treatments induce large time-dependent increases in basal neuronal firing in the brainstem nuclei for serotonin in the dorsal raphe nucleus (89). Thus, chronic VNS was associated with increased extracellular levels of serotonin in the dorsal raphe (90).

Several lines of evidence suggest that NE is a neurotransmitter of major importance in the pathophysiology and treatment of depressive disorders (91). Thus, experimental depletion of NE in the brain led to a return of depressive symptoms after successful treatment with NE antidepressant drugs (91). The LC contains the largest population of noradrenergic neurons in the brain and receives projections from NTS, which, in turn, receives afferent input from the vagus nerve (92). Thus, VNS leads to an enhancement of the firing activity of NE neurons (93), and consequently, an increase in the firing activity of serotonin neurons (94). Thus, VNS was shown to increase the NE concentration in the prefrontal cortex (95). The pharmacologic destruction of noradrenergic neurons resulted in the loss of antidepressant VNS effects (96).

In case of DA, it has been shown that the short-term effects (14 days) (94) and the long-term effects (12 months) (97) of VNS in treatment of resistant major depression may lead to brainstem dopaminergic activation. DA is a catecholamine that to a large extent is synthesized in the gut and plays a crucial role in the reward system in the brain (98).

Further, beneficial effects of VNS might be exerted through a monoamine-independent way. Thus, VNS treatments might result in dynamic changes of monoamine metabolites in the hippocampus (93) and several studies reported the influence of VNS on hippocampal neurogenesis (99, 100). This process has been regarded as a key biological process indispensable for maintaining the normal mood (101).

Serotonin is also an important neurotransmitter in the gut that can stimulate peristalsis and induce nausea and vomiting by activating the vagus nerve. In addition, it is essential for the regulation of vital functions, such as appetite and sleep, and contributes to feelings of well-being. To 95%, it is produced by enterochromaffin cells, a type of neuroendocrine cell which reside alongside the epithelium lining the lumen of the digestive tract (102). Serotonin is released from enterochromaffin cells in response to mechanical or chemical stimulation of the gastrointestinal tract which leads to activation of 5-HT3 receptors on the terminals of vagal afferents (103). 5-HT3 receptors are also present on the soma of vagal afferent neurons, including gastrointestinal vagal afferent neurons, where they can be activated by circulating 5-HT. The central terminals of vagal afferents also exhibit 5-HT3 receptors that function to increase glutamatergic synaptic transmission to second order neurons of the nucleus tractus solitarius within the brainstem. As a result, interactions between the vagus nerve and serotonin systems in the gut and in the brain appear to play an important role in the treatment of psychiatric conditions.

## Vagus-Related Treatment of Depression

### Basic Pathophysiology of Depression

Major depressive disorder ranks among the leading mental health causes of the global burden of disease (104). With a lifetime prevalence of 1.0% (Czech Republic) to 16.9% (US) (105), the cost of depression poses a significant economic burden to our society (106). The pathophysiology of depression is complex and includes social environmental stress factors; genetic and biological processes, such as the overdrive of the HPA axis, inflammation (31), and disturbances in monamine neurotransmission as described above (91). For example, a lack of the amino acid tryptophan, which is a precursor to serotonin, can induce depressive symptoms, such as depressed mood, sadness, and hopelessness (86).

The overdrive of the HPA axis is most consistently seen in subjects with more severe (i.e., melancholic or psychotic) depression, when the cortisol feedback inhibitory mechanisms are impaired, contributing to cytokine oversecretion (107). It has been shown that chronic exposure to elevated inflammatory cytokines can lead to depression (108). This might be explained by the fact that cytokine overexpression leads to a reduction of serotonin levels (109). In line with that, treatment with anti-inflammatory agents has the potential to reduce depressive symptoms (110). In line, IBD are important risk factor for mood and anxiety disorders (111), and these psychiatric conditions increase the risk of exacerbation of IBD (112).

### VNS in Depression

A European multicenter study demonstrated a positive effect of VNS on depressive symptoms, in patients with treatment-resistant depression (113). The application of VNS over a period of 3 months resulted in a response rate of 37% and a remission rate of 17%. After 1 year of treatment, the response rate reached 53% and the remission rate reached 33%. A meta analysis that compared the application of VNS to the usual treatment in depressed patients showed a response rate of approximately 50% in the acute phase of the disease and a long-term remission rate of 20% after 2 years of treatment (114). Several other studies also demonstrated an increasing long-term benefit of VNS in recurrent treatment-resistant depression (84, 85, 115). Further, a 5-year prospective observational study which compared the effects of treatment as usual and VNS as adjunctive treatment with treatment as usual only in treatment-resistant depression, showed a better clinical outcome and a higher remission rate in the VNS group (116). This was even the case in patients with comorbid depression and anxiety who are frequent non-responders in trials on antidepressant drugs. It is important to note that all these studies were open-label and did not use a randomized, placebo-controlled study design.

Patients with depression have elevated plasma and cerebrospinal fluid concentrations of proinflammatory cytokines. The benefit of VNS in depression might be due to the inhibitory action on the production of proinflammatory cytokines (117) and marked peripheral increases in anti-inflammatory circulating cytokines (118). Further, improvement after VNS was associated with altered secretion of CRH, thus preventing the overdrive the HPA axis (119). Altered CRH production and secretion might result from a direct stimulatory effect, transmitted from the vagus nerve through the NTS to the paraventricular nucleus of the hypothalamus. Finally, VNS has been shown to inhibit peripheral blood production of TNF-α which is increased in clinical depression (10).

### Influence of Nutrition Depressive Symptoms

The gut microbiota is the potential key modulator of the immune (120) and the nervous systems (121). Targeting it could lead to a greater improvement in the emotional symptoms of patients suffering from depression or anxiety. There is growing evidence that nutritional components, such as probiotics (122, 123), gluten (124), as well as drugs, such as anti-oxidative agents (125) and antibiotics (126), have a high impact on vagus nerve activity through the interaction with the gut microbiota and that this effect varies greatly between individuals. Indeed, animal studies have provided evidence that microbiota communication with the brain involves the vagus nerve and this interaction can lead to mediating effects on the brain and subsequently, behavior (127). For example, *Lactobacillus*-species have received tremendous attention due to their use as probiotics and their health-promoting properties (128). Bravo et al. (129) demonstrated that chronic treatment of mice with *Lactobacillus rhamnosus* (strain JB-1) caused a reduction in stress-induced corticosterone levels and in anxiety-like and depression-like behavior (129). It has been shown that chronic treatment with *L. rhamnosus* (JB-1) induced region-dependent alterations in GABA(B1b) mRNA in the brain with increases in cortical regions (cingulate and prelimbic) and concomitant reductions in expression in the hippocampus, amygdala, and LC. In addition, *L. rhamnosus* (JB-1) reduced GABA(Aα2) mRNA expression in the prefrontal cortex and amygdala, but increased GABA(Aα2) in the hippocampus (129), which counteracts the typical pathogenesis of depressive symptoms: lack of prefrontal control and overactivity of subcortical, anxiogenic brain regions. Importantly, *L. rhamnosus* (JB-1) reduced stress-induced corticosterone and anxiety- and depression-related behavior. This is not surprising, since alterations in central GABA receptor expression are implicated in the pathogenesis of anxiety and depression (130, 131). The antidepressive and anxiolytic effects of *L. rhamnosus* were not observed in vagotomized mice, identifying the vagus as a major modulatory constitutive communication pathway between the bacteria exposed to the gut and the brain (129). In line with that, in a model of chronic colitis associated to anxiety-like behavior, the anxiolytic effect obtained with a treatment with *Bifidobacterium longum*, was absent in mice that were vagotomized before the induction of colitis (132).

In humans, psychobiotics, a class of probiotics with anti-inflammatory effects might be useful to treat patients with psychiatric disorders due to their antidepressive and anxiolytic effects (133). Differences in the composition of the gut microbiota in patients with depression compared with healthy individuals have been demonstrated (134). Importantly, the fecal samples pooled from five patients with depression transferred into germ-free mice, resulted in depressive-like behavior.

### Influence of Relaxation Techniques on Depressive Symptoms

It has been shown that self-generated positive emotions *via* loving-kindness meditation lead to an increase in positive emotions relative to the control group, an effect moderated by baseline vagal tone (135). In turn, increased positive emotions produced increases in vagal tone, which is probably mediated by increased perceptions of social connections. Individuals suffering from depression, anxiety, and chronic pain have benefited from regular mindfulness meditation training, demonstrating a remarkable improvement in symptom severity (9).

Controlled studies have found yoga-based interventions to be effective in treating depression ranging from mild depressive symptoms to major depressive disorder (MDD) (136). Some yoga practices can directly stimulate the vagus nerve, by increasing the vagal tone leading to an improvement of autonomic regulation, cognitive functions, and mood (137) and stress coping (138). The proposed neurophysiological mechanisms for the success of yoga-based therapies in alleviating depressive symptoms suggest that yoga breathing induces increased vagal tone (139). Many studies demonstrate the effects of yogic breathing on brain function and physiologic parameters. Thus, Sudarshan Kriya Yoga (SKY), a breathing-based meditative technique, stimulates the vagus nerve and exerts numerous autonomic effects, including changes in heart rate, improved cognition, and improved bowel function (140). During SKY, a sequence of breathing techniques of different frequencies, intensities, lengths, and with end-inspiratory and end-expiratory holds creates varied stimuli from multiple visceral afferents, sensory receptors, and baroreceptors. These probably influence diverse vagal fibers, which in turn induce physiologic changes in organs, and influence the limbic system (140). A recent study showed that even patients who did not respond to antidepressants showed a significant reduction of depressive and anxiety symptoms compared to the control group after receiving an adjunctive intervention with SKY for 8 weeks (141).

Iyengar yoga has been shown to decreased depressive symptoms in subjects with depression (142). Iyengar yoga is associated with increased HRV, supporting the hypothesis that yoga breathing and postures work in part by increasing parasympathetic tone (143).

## Vagus-Related Treatment of PTSD

### Pathophysiology of PTSD

Posttraumatic stress disorder is an anxiety disorder that can develop after trauma and is characterized by experiencing intrusive memories, flashbacks, hypervigilance, nightmares, social avoidance, and social dysfunctions (144). It has a lifetime prevalence of 8.3% using the definition for DSM-5 (145). The symptoms of PTSD can be classified into four clusters: intrusion symptoms, avoidance behavior, cognitive and affective alterations, and changes in arousal and reactivity (146). People who suffer from PTSD tend to live as though under a permanent threat. They exhibit fight and flight behavior or a perpetual behavioral shutdown and dissociation, with no possibility of reaching a calm state and developing positive social interactions. Over time, these maladaptive autonomic responses lead to the development of an increased risk for psychiatric comorbidities, such as addiction and cardiovascular diseases (147).

Posttraumatic stress disorder symptoms are partly mediated by the vagus nerve. There is evidence for diminished parasympathetic activity in PTSD, indicating an autonomic imbalance (148). The vagal control of heart rate *via* the myelinated vagal fibers varies with respiration. Thus, the vagal influence on the heart can be evaluated by quantifying the amplitude of rhythmic fluctuations in heart rate—respiratory sinus arrhythmia (RSA). A recent study has demonstrated a reduced resting RSA in veterans with PTSD (149). Further, patients with PTSD have been shown to have lower high-frequency heart rate variability than healthy controls (150).

Continuous expression of emotional symptoms to conditioned cues despite the absence of additional trauma is one of the many hallmarks of PTSD. Behavioral therapies employed to treat PTSD rely on helping the patient to gradually reduce her/his fear of this cue over time. Thus, exposure-based therapies are considered the gold standard of treatment for PTSD (151). The goal of exposure-based therapies is to replace conditioned associations of the trauma with new, more appropriate associations which compete with fearful associations. Studies have shown that PTSD patients exhibit deficient extinction recall along with dysfunctional activation of the fear extinction network (152, 153). This network includes the vmPFC, the amygdala, and the hippocampus. It is highly important for the contextual retrieval of fear memories after extinction (154).

Posttraumatic stress disorder symptom severity and structural abnormalities in the anterior hippocampus and centromedial amygdala have been associated (155). There is evidence for increased activation of the amygdala in humans and rodents during conditioned fear (156). The amygdala and the vmPFC have reciprocal synaptic connections (157). Indeed, under conditions of uncertainty and threat, the PFC can become hypoactive leading to a failure to inhibit overactivity of the amygdala with emergence of PTSD symptoms, such as hyperarousal and re-experiencing (158). Further, in response to stressful stimuli as fearful faces, patients with PTSD showed a higher activation of the basolateral amygdala during unconscious face processing compared to healthy controls as well as patients with panic disorder and generalized anxiety disorder (159).

The hippocampus is also a crucial component of the fear circuit and implicated in the pathophysiology of PTSD. Patients with PTSD show a reduced hippocampal volume that is associated with symptom severity (160). The hippocampus is a key structure in episodic memory and spatial context encoding. Hippocampal damage leads to deficits in context encoding in humans as well as rodents. The neural circuit consisting of the hippocampus, amygdala, and vmPFC is highly important for the contextual retrieval of fear memories after extinction (154). Impairment of hippocampal functioning, resulting dysfunctional context generalization in patients with PTSD, might cause patients to re-experience trauma-related symptoms (161).

### VNS in PTSD

Vagus nerve stimulation has shown promise as therapeutic option in treatment-resistant anxiety disorders, including PTSD (8). Chronic VNS has been shown to reduce anxiety in rats (96) and improve scores on the Hamilton Anxiety Scale in patients suffering from treatment-resistant depression (8). When stimulated, the vagus nerve sends signals to the NTS (162) and the NTS sends direct projections to the amygdala and the hypothalamus. Further, VNS increases the release of NE in basolateral amygdala (163) as well as the hippocampus and cortex (93). NE infusion in the amygdala results in better extinction learning (164). Thus, VNS could be a good tool to increase extinction retention. For example, in rats, extinction paired with VNS treatment can lead to remission of fear and improvements in PTSD-like symptoms (151). Further, VNS paired with extinction learning facilitates the plasticity between the infralimbic medial prefrontal cortex and the basolateral complex of the amygdala to facilitate extinction of conditioned fear responses (165). Additionally, VNS may also enhance extinction by inhibiting activity of the sympathetic nervous system (119). It is possible that an immediate VNS-induced reduction in anxiety contributes to VNS-driven extinction by interfering with the sympathetic response to the CS, thus breaking the association of the CS with fear. However, there is need for randomized controlled trials to approve these observations.

One of the most consistent neurophysiological effects of VNS is decreasing the hippocampal activity, possibly through enhancement of GABAergic signaling (166). As described above, the hippocampus is a crucial component of the fear circuit, since it is a key structure in episodic memory and spatial context encoding. Decreased hippocampal activity after VNS has been reported in a number of other studies in other conditions such as depression (77, 167) or schizophrenia (168).

### Positive Influence of Nutritive Components on PTSD

Emerging research suggests that probiotics may have the potential to decrease stress-induced inflammatory responses, as well as associated symptoms. An exploratory study that investigated the microbiome of patients with PTSD and trauma exposed controls revealed a decreased existence of three bacteria strains in patients with PTSD: Actinobacteria, Lentisphaerae, and Verrucomicrobia that were associated with higher PTDS symptom scores. These bacteria are important for immune regulation and their decreased abundance could have contributed to a dysregulation of the immune system and development of PTSD symptoms (169). A study using a murine model of PTSD (170) has demonstrated that immunization with a heat-killed preparation of the immunoregulatory bacterium *Mycobacterium vaccae* (NCTC 11659) induced a more proactive behavioral response to a psychosocial stressor (171). Studies performed in healthy volunteers have shown that the administration of different probiotics were associated with an improved well-being (172–174), as well as a decrease in anxiety and psychological distress (174, 175). These findings are all preliminary. There is an urgent need for well-designed, double-blind, placebo-controlled clinical trials aimed at determining the effect of bacterial supplements and controlled changes in diet on psychological symptoms and cognitive functions in patients with PTSD.

### Positive Influence of Meditation and Yoga on PTSD

There is clinical evidence for the efficacy of mindfulness-based stress reduction (MBSR) in the treatment of PTSD (176–178). During MBSR, slow breathing and long exhalation phases lead to an increase in parasympathetic tone (179). In addition, clinical studies have demonstrated the effectiveness of yoga as a therapeutic intervention for PTSD and dissociation through a downregulation of the stress response (180–182). Yoga practices also decreased symptoms in PSTD after natural disasters (183, 184). Yoga-responsive anxiety disorders, including PTSD, go together with low HRV and low GABA activity (139). The interactions of the PFC, hippocampus, and amygdala in conjunction with inputs from the autonomous nervous system and GABA system provide a network through which yoga-based practices may decrease symptoms (185). There are indications that impaired extinction of conditioned fear in PTSD is associated with decreased vmPFC control over amygdala activity (157). PFC activation associated with increased parasympathetic activity during yoga could improve inhibitory control over the amygdala *via* PFC GABA projections, decreasing amygdala overactivity, and reducing PTSD symptoms.

## Vagus-Related Treatment in Inflammatory Disease

### Pathophysiology IBD

Inflammatory bowel disease comprises mainly two disorders: ulcerative colitis (UC) and Crohn’s disease (CD) (186). The hallmark of IBD is chronic, uncontrolled inflammation of the intestinal mucosa. Symptoms are characterized by abdominal pain, diarrhea, fever, weight loss, and extraintestinal (skin, eyes, joints) manifestations. In CD, the predominant symptoms are diarrhea, abdominal pain, and weight loss, whereas in UC diarrhea is the main symptom, often accompanied by rectal bleeding (187).

Inflammatory bowel disease affects about 1.5 million persons in the USA and 2.2 million in Europe (188), and about 20% of IBD patients have a positive family history (189). In addition, industrialization led to marked increases in IBD prevalence rates in Asia (190). There is increasing evidence that environmental risk factors, including infections, Western diet and food additives, air and water pollution, drugs (antibiotics, hormones), and psychosocial stress work in concert with genetic factors (more than 250 genetics factors have been consistently identified) in the pathogenesis of IBD, finally leading to an abnormal immune response to microbial exposure (191, 192). What distinguishes IBD from inflammatory responses seen in the normal gut is an inability to downregulate inflammatory responses, like it happens when intestine becomes inflamed in response to a potential pathogen. Thus, in individuals with IBD inflammation is not downregulated, the mucosal immune system remains chronically activated, and the intestine remains chronically inflamed (191). During inflammation, proinflammatory cytokines (IL-1β, IL-6, TNF-α) released from the intestinal mucosa activate VN afferents that terminate in the NTS (188), then relaying visceral information to activate the HPA axis. Moreover, an anti-inflammatory role of vagus efferents through the CAIP has been reported (188). As stated earlier, ACh released at the distal end of VN efferents decreases the production of proinflammatory cytokines, such as TNF-α (188). The overexpression of the TNF-α may present a curical step in the development of IBD (193).

### VNS in IBD

Vagus nerve stimulation attenuates the systemic inflammatory response to endotoxin (73) and intestinal inflammation (194). The VNs also indirectly modulates immune activity of the spleen through connections with the splenic sympathetic nerve (13). In rats with colonic inflammation, the 3 h long daily VNS for a period of 5 days led to a reduction in inflammatory markers and an improvement in symptoms of colitis (195).

Vagus nerve stimulation should be of interest in other inflammatory diseases, such as rheumatoid arthritis, another TNF-α-mediated disease. In patients with rheumatoid arthritis, a study that demonstrated an improvement of symptoms in the early and late stages of the disease through 1–4 min of VNS daily (10). This study was also the first to show that VNS inhibits the production of TNF-α and other cytokines in humans by stimulating the inflammatory reflex, leading to an improvement of symptom severity. These data argue for an anti-inflammatory role of the vagus nerve and provide potential therapeutic applications for patients with IBDs (18, 195, 196).

### Positive Influence of Nutritive Components on IBD

Mechanistically, the role that inflammation plays in the onset and perpetuation of psychiatric symptoms has garnered increased attention (197). The increase of dysfunctional immunological responses in modern urban societies are posited to be at least in part associated with reduced exposure to commensal and environmental microorganisms that normally prime immunoregulatory circuits and suppress inappropriate inflammation (198). The intestinal bacterial flora is thought to be an important factor in the development and recurrence of IBD and various attempts have been made to modify the flora with probiotics. In animals with experimental colitis orally or rectally administered lactobacilli have yielded improvements. For example, *Lactobacillus plantarum* 299V prevented the onset of disease and reduced established colitis (199). Further, a multispecies probiotic (VSL#3) given to mice with established colitis normalized gut barrier function, reduced proinflammatory cytokines, and lessened histological disease (200). In humans, *Lactobacillus casei* GG improved symptoms in children with moderately active CD (201). In addition, a combination of probiotics with *Saccharomyces boulardii, Lactobacillus*, and VSL#3 showed slight improvements of CD symptoms (202). These data are preliminary and need confirmation by future studies. So far, no probiotic treatments have been officially recommended for the treatment of CD (203).

In UC, there is reliable evidence for VSL#3 to be beneficial in the treatment of mildly active pouchitis (204). *E. coli* Nissle, part of VSL#3, may be as effective as the drug mesalamine in maintaining remission (205).

### Positive Influence of Hypnotherapy, Meditation, and Yoga in IBD

An increasing number of studies have shown benefits with relaxation-related treatment of IBD. For example, a randomized controlled trial of a relaxation-training intervention compared to a control group has shown decrease in pain as well as decreased anxiety levels and improvements in quality of life (206). Also mindfulness-based therapy (207), a comprehensive mind-body program (208), meditation (209), mind-body alternative approaches (210), yoga (211), and relaxation response-based mind-body interventions (212) have shown to be beneficial for IBD patients. In addition, hypnotherapy, which increases vagal tone (213), has been effective in the treatment of IBD (12).

## Conclusion

The interaction between the gut and the brain is based on a complex system that includes not only neural but also endocrine, immune, and humoral links.

The vagus nerve is an essential part of the brain–gut axis and plays an important role in the modulation of inflammation, the maintenance of intestinal homeostasis, and the regulation of food intake, satiety, and energy homeostasis. An interaction between nutrition and the vagus nerve is well known, and vagal tone can influence food intake and weight gain.

Moreover, the vagus nerve plays an important role in the pathogenesis of psychiatric disorders, obesity as well as other stress-induced and inflammatory diseases.

Vagus nerve stimulation and several meditation techniques demonstrate that modulating the vagus nerve has a therapeutic effect, mainly due to its relaxing and anti-inflammatory properties.

Extinction paired with VNS is more rapid than extinction paired with sham stimulation. As it is currently approved by the Federal FDA for depression and seizure prevention, VNS is a readily available and promising adjunct to exposure therapy for the treatment of severe anxiety disorders.

Vagus nerve stimulation is an effective anticonvulsant device and has shown in observational studies antidepressant effects in chronic treatment-resistant depression. Because the vagus nerve sends information to brain regions is important in the stress response (LC, orbitofrontal cortex, insula, hippocampus, and amygdala), this pathway might be involved in perceiving or manifesting various somatic and cognitive symptoms that characterize stress-related disorders.

Psychotropic drugs, such as serotonin reuptake inhibitors, have effects on both the brain and the gastrointestinal tract and consequently should be understood as modulators of the brain–gut axis.

Research investigating the interaction between nutritive factors, somatic factors, such as heart rate, psychological and pharmacological treatments, and vagal activity has the potential to lead to integrative treatment options that incorporate VNS, nutritional approaches, drugs, and psychological interventions, such as mindfulness-based approaches, which can be tailored to the needs of the individual patient.

## Author Contributions

SB, AK and GR reviewed literature and wrote this paper. GH outlined structure of this paper, reviewed literature, and wrote this paper.

## Conflict of Interest Statement

The authors declare that the research was conducted in the absence of any commercial or financial relationships that could be construed as a potential conflict of interest. The reviewer JD and handling editor declared their shared affiliation.
